# Supplementary figures and images for: The Effect of High-Intensity Interval Training Type on Body Fat Percentage, Fat and Fat-Free Mass: A Systematic Review and Meta-Analysis of Randomized Clinical Trials
Source: J Clin Med. 2023 Mar 15;12(6):2291. doi: 10.3390/jcm12062291 (PMC10054577; doi:10.3390/jcm12062291)

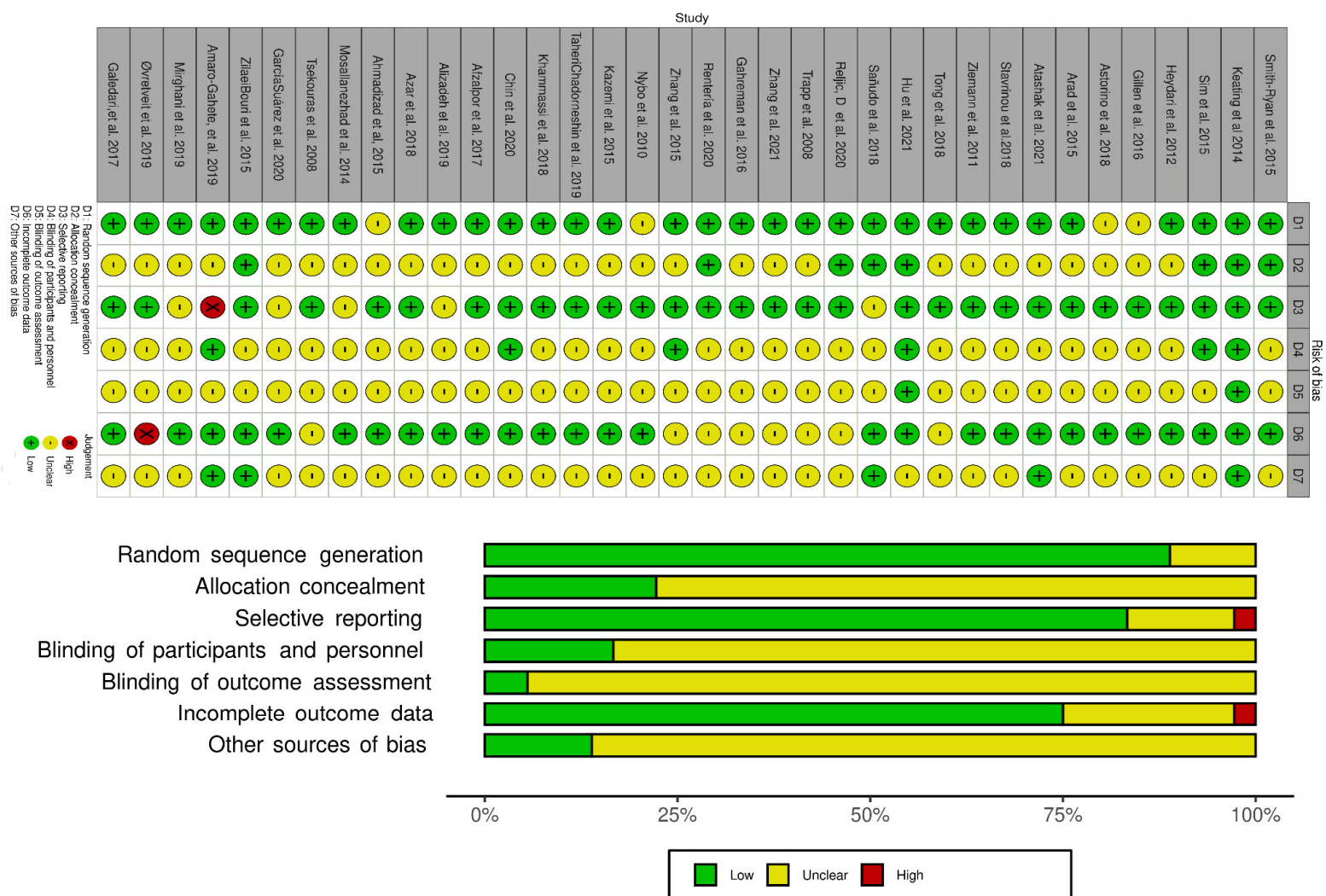

Supplementary File S1. Results of the quality assessment

Supplement: Supplementary file 1 [file jcm-12-02291-s001.zip › jcm-2196683-supplementary.pdf]
